# On the Use of Chloroform

**Published:** 1854-04

**Authors:** 


The *Medical Examiner* for December, gives from the London Medical Times, account of three cases of death from chloroform, occurring during the month of October. It is thought by a correspondent of the Times, that in the hospital practice great error prevails in its administration, and that "sufficient precautions are not used to allow a free admixture of atmospheric air with the anæsthetic."

Since then a case has been reported in the Buffalo Medical Journal for December, by Dr. T. K. DeWolf, of Chester. Mass., bearing date of October 3d, 1853; and another is recorded in the January No. of the New Orleans Medical and Surgical Journal for January, as occurring in the Charity Hospital. The report has the initials of Dr. Hester, but is without date. These fatal cases naturally re-awaken the alarms of the profession.—*South. Jour. of the Med. & Physical Sciences.*

And yet, after all these multiplied evidences of its capacities for mischief, there are those who continue to maintain that it is a harmless agent. Like almost every other therapeutic agent, the very fact of its great potency for good is an evidence of its capacity for evil.—There is much propriety and truth in what Dr. Meigs has said with regard to the use of this article, the opinions of the celebrated Simpson to the contrary notwithstanding. We solemnly protest against the indiscriminate use of this anæsthetic, and particularly to the slovenly and unskillful use of it, and this position we have ever occupied since its discovery. The time has gone by, we trust, for charlatans to manufacture capital out of its use, for the secular journals have warned community by repeated publications of its fatal powers in very many instances. Even after every precaution has been used, by the most scientific, deaths have occurred, which fact emphatically shows that the utmost caution should be exercised, and this too by the most skillful.

Like every other valuable remedy it may be abused, and this abuse will bring it into disrepute with the multitude, and the most inveterate prejudices will be awakened against *it* and those who use it. The time was when calomel was thought to be a *panacea* for the cure of all physical ills, and was prescribed in almost all cases; but after a time it beeame odious in consequence of the evils arising out of its indiscriminate exhibition, and a war was raised against it, which resulted in the rearing up a hydra-headed system of quackery, on whose floating banner were mottoes inscribed in burning letters of indignation against mercurial and other mineral "*pisons.*" The war cry will soon be raised against chloroform, the timid will not dare to use it even as a dernier resort, and the credulity of the people will be made to pander to the greed of a brawling band of wooden-headed hypocrites, whose only capacity is to mislead and deceive.

The profession may well charge to its own imprudence and want of discretion the growth of these *fungi*, and we think that the past contains a pertinent warning for the future. We would grieve to witness a tirade against chloroform, or any other anæsthetic agent, because of its value in those cases in which it should be resorted to from the imperative requirements for its use, but in which, because of the deep-seated prejudices against it, the profession would be prohibited its use, and humanity its benefits. To avoid such a result then, and because of the reports of fatal cases, in almost every journal which comes to our hands, we would urge again the greatest caution, circumspection and judgment in its use. The best interests of the profession in the future, and of humanity, alike demand this declaration at our hands, and we sincerely hope that the days of *hobby-mania* will be of short duration, and that every new discovery will be treated with that jealous suspicion which will ensure the most careful examination of its properties and effects upon the animal economy.

